# Synthetic biology in the German press: how implications of metaphors shape representations of morality and responsibility

**DOI:** 10.1186/s40504-018-0079-9

**Published:** 2018-06-24

**Authors:** Martin Döring

**Affiliations:** Institute of Geography, University of Hamburg, Bundesstraße 55, 20146 Hamburg, Germany

**Keywords:** Synthetic biology, Metaphor, Moral implications, German press coverage

## Abstract

Synthetic biology (SynBio) represents a relatively young field of research which has developed into an important scientific endeavour. Characterised by a high degree of interdisciplinary work crossing disciplinary boundaries, such as biology, mathematics and engineering, SynBio has been, since its beginning, devoted to creating new biological functions, metabolic pathways or even minimal organisms. Although its often-articulated aim of developing new forms of life has so far not been archived, SynBio nowadays represents a well-established biotechnological approach and it has also attracted public concern, especially since Craig Venter’s work on Mycoplasma Mycoides JCVI-syn1.0. Taking these developments as a starting point, the paper empirically investigates the metaphorical representations of SynBio in two leading German media publications, the daily newspaper *Die Frankfurter Allgemeine Zeitung* and the weekly magazine *Der Spiegel* between 2000 and 2010. Using a novel combination of metaphor and co-occurrence analysis, the paper engages in a systematic examination of implicit moral implications inherent in linguistic images permeating this news coverage. It demonstrates a method of how media-metaphorical representations and their moral implications of SynBio could analytically be revealed and analysed. In doing so, it aims at contributing to empirical ethical analyses of the news coverage on SynBio in particular and offers an approach that methodologically adds to literature on responsible language use, which is emerging in science and technology studies and ethical analyses of new technologies.

## Synthetic biology: an emerging and consolidating field of research

Synthetic Biology (SynBio) is a relatively young field of research (Endy 2005), closely linked to biotechnology, systems biology and metabolic engineering (Nesbet 2016). After 15 years of existence (De Lorenzo and Danchin 2008; Nisbet and Lewenstein 2002), it can now be regarded as a scientifically established discipline with its own journals, conferences, research centres and curricula at universities all over the world. The disciplinary rationale of SynBio is twofold, namely: “the design and construction of new biological parts, devices and systems as well as the redesign of existing parts” (Porcar and Peretó 2012: 79).

Questions continue to be asked as to whether SynBio is a new scientific discipline in a biotechnological field of research where proteomics, systems biology (Döring et al. 2015) and metabolic engineering are comparable ambitious endeavours and whether Synbio’s engineering approach makes it a unique discipline or not (Keller 2002). Interestingly, studies from the field of the sociology of science and science and technology studies frame SynBio as a scientific discipline. Here, SynBio is conceived as a post-genomic engineering discipline that aims at making biology “less qualitative and descriptive and more quantitative and predictive” (Calvert and Fujimura 2009: 48). This shift, Calvert and Fujimura assume, exhibits a conceptual and methodological attempt to make biology a hard(er) science and develop a high degree of sophistication by including a quantitatively based rationale derived from computer science, mathematics and modelling. This has theoretical and methodological repercussions for biology, biotechnology, biomedical research, science-policy, the economy and society in general.

The increasing scientific relevance of SynBio is also mirrored in the number of scientific publications found on Pubmed-Reminer (search term ‘Synthetic Biology’) which remained until 2008 at an average of approx. 60 papers per year, but has risen to 827 in 2017. The same holds true for scientific reviews – an important type of text written by experts reviewing research undertaken and assessing the future of a discipline. Here we find a steep increase between 2011 (57 reviews) and 2016 (231 reviews), only slowing down in 2017 to 198 reviews. This clearly indicates that a scientific narrative is woven around SynBio which “shapes the literature of a field into a story in order to enlist the support of readers to [...] [corroborate] this story” (Meyers 1991: 45). These numbers have, however, to be treated with caution as they do not indicate whether there is a scientific revolution taking place or whether rising figures, in terms of publication output, indicate a fashionable use of the term SynBio for strategic purposes.

Furthermore, it should be stressed that many targets set by scientists working in the field of SynBio have not been achieved to date while others have materialised and attracted considerable scientific, political and public attention. This was the case in the development of Artemisinin, an anti-malaria drug extracted from engineered yeast and even more so in the production of the artificial organism Mycoplasma Mycoides JCVI-syn1.0 (Balmer and Herreman 2009: 221). Additionally, conceptual issues such as the principles of hierarchical abstraction or modularity (Serrano 2007: 1) or the integration of the so-called chassis and the programme cause considerable problems (Dachin 2012).

At a large press conference, Craig Venter publicly announced that the merging of synthetic sequences with sequences taken from another bacterium had been successful and proclaimed that the shift from biological reproduction of life, to the technological production of life, had been achieved by him and his research team (Cserer and Seiringer 2009). Such proclaimed scientific revolutions are in many cases accompanied by intensive media coverage and can lead to worries about the release of artificially generated life forms into the environment (Balmer and Martin 2008). Venter’s development of Mycoplasma Mycoides JCVI-syn1.0 was indeed accompanied by rapid and considerable media coverage semantically revolving around the metaphor of “playing god in Frankenstein’s footsteps” (van Belt 2009: 257). It was interesting to observe how the creation of a supposedly artificial form of life caused considerable public concern about its possible ecological impact, but also how such an exceptional event was metaphorically and linguistically represented.

All these observations form the starting point of the present paper which aims at providing a methodologically systematic and empirical analysis of moral implications inherent in the metaphors that permeated the German newspaper coverage of SynBio in the seminal daily newspaper *Die Frankfurter Allgemeine Zeitung* and the renowned weekly magazine *Der Spiegel*. This article aims to methodologically and empirically complement existing philosophical and bioethical reflections in the areas of biotechnological innovation, biomedical developments (Nordgren 1998), systems biology (Döring et al. 2015), synthetic biology (Boldt 2016), and science and technology studies (Komduur et al. 2009). It differs from this literature insofar as it proposes a novel practical approach for studying metaphorical mapping processes from an ethical point of view. This is important because in the mapping process arguments and values are cognitively transferred from one domain of knowledge to another: the “production of certain [metaphorical] mappings is an act of ethical discourse” (Balmer and Herreman 2009: 221), which includes unnoticed or even hidden moral implications. This requires conscious reflection in terms of a responsible use of metaphors which “are often used to help readers to connect to scientific results” (Kueffer and Larson 2014) and developments.

In the following, I will first provide an overview of, and theoretically engage with, research undertaken on metaphor in linguistics and in the fields of science and technology studies with a focus on studies investigating images in the areas of genetics, genomics and synthetic and systems biology. Against this background, I will explain the approach taken for studying metaphors (Schmitt 2011). I will then explicate my method of analysing metaphorical mappings with the aid of a co-occurrence analysis. The article will show how this analysis allows researchers to perform an empirically grounded analysis of hidden implications, moral or otherwise, in metaphorical mappings. Illustrating this approach, I will analyse four main metaphorical concepts semantically permeating the German news coverage. In the final section, I will summarise the theoretical, methodological and empirical implications of my findings and reflect on the fact that a qualitatively improved science communication can contribute to the responsible use of metaphors.

## Metaphor and synthetic biology: theoretical dimensions for analysing moral implications

Nowadays it is a truism to state that metaphors pervade science (Brown 2008) and are creatively applied in scientific thinking (Katherndahl 2014). Numerous scholars in different areas of research have studied the constitutive role of metaphors for scientific thinking and the development of scientific concepts, theories and methods.

The works by the philosophers Black (1962) and Blumenberg (1960) were among the first to develop coherent approaches and tools for analysing metaphors – not only – in scientific thought. Gentner and Jeziorski (1993) have investigated the formation of scientific work from a historical point of view tracing the conceptual steps taken from metaphor to analogy. They reveal important structural mappings in the works of scientists, such as Sadi Carnot, Robert Boyle or Paracelus (Gentner and Jeziorski 1993: 448), showing how metaphorical mappings and analogies informed the development of scientific theories. Hesse, in turn, (1966; 1970) applied Max Black's (1962) and Ivor Richard’s (1936) interaction theory of metaphor and unravelled the explanatory power of metaphor in the context of the formation of analogical models in science.

This research inspired Kay (2000) who investigated central metaphors in genetics such as “books”, “maps” and “information theory”, which all contributed to establishing the scientific rationale of molecular biology (Kay 1997).

Later works by Maasen and Weingart (2000), Larson (2011) and Döring et al. (2015) are among the few who provide a discussion of different theories of metaphor, integrate them into the sociology of science and science and technology studies, and provide a methodological approach for exploring metaphorically driven knowledge dynamics in science. This also applies to the work of Nerlich and Hellsten (2005, 2011) who also performed comprehensive empirical research on metaphors in genetics, genomics and synthetic biology, especially news coverage.

Even though a lot of research has been undertaken on different levels of media framings, the production and reception of news in different countries (see for example Ancillotti and Eriksson 2016), a critical reflection, theoretical integration and methodological elaboration of philosophical and philological research is still missing. This applies in particular to metaphor theories proposed by Blumenberg (1960) Black (1962), Weinrich (1976), Lakoff and Johnson (1980), Lakoff (1987), Johnson (1987) and extended by others (see Jäkel 1997: 141–146; Döring 2005: 25–122).

Most of the social science research focusing on SynBio is rooted in media and communication sciences, philosophy and history. Hence, methods such as Grounded Theory or approaches stemming from qualitative research are used to establish and analyse media corpora (Gschmeidler and Seiringer 2012) for the analysis of metaphors (Nerlich and Hellsten 2011).

However, studies on the use and role of metaphor (Cserer and Seiringer 2009) often only partly engage with the theoretical dimensions of systematic research on metaphor (Schmitt 2011). This has led to analytical imprecision (Dabrock 2009), mixing linguistic and conceptual imagery and questions revolving around the relevance of certain types of metaphors in the news coverage. The criticism applies both to Keller’s (2002) seminal book on metaphors and models used in biology and to an article by Boudry and Pigliucci (2013), which provides an interesting, critical and historical investigation of engineering metaphors in SynBio without developing a basic notion of metaphor. The section on metaphor and worldviews in Boldt’s (2016) edited volume follows a comparable rationale: the philosophical analyses undertaken here are interesting but a closer engagement with the theories of metaphor would have probably added precision to the ethical assessment of SynBio.

Hellsten and Nerlich (2008) and O’Keefe et al. (2015) for example are among the few who perform a systematic analysis that not only includes the development of a comprehensive media corpus and the application of social science methods, but also engage deeply with theories and methods research on metaphor. They rely on five aspects of metaphor which they argue are of analytical importance. First, *metaphors are ubiquitous* phenomena that pervade all discourses, be they scientific, political or public. They are a basic ingredients of human thinking and not just artistic or poetic decorations (Lakoff and Johnson 1980). In this respect conventionalised metaphors are more important than creative ones as they semantically structure and shape everyday thinking and talking, often pass unnoticed but work unconsciously (Lakoff and Johnson 1980: 139). Second, *metaphors possess a focusing function* (Blumenberg 1960: 75). They highlight certain semantic aspects of a topic or discursive domain while hiding others (Jäkel 1997: 42). This can strongly constrain how we conceptualise the world but also open-up the possibility to question current conceptualisations and to develop alternative ones (Lakoff and Johnson 1980: 96). Thus – and thirdly – *metaphors are creative devices* (Weinrich 1976: 175) for the production and shaping of meaning: they can be used to generate or explore alternative meanings that go beyond existing meanings (Lakoff and Turner 1989:80). This heuristic ability to restructure thought patterns (Jäkel 1997: 35) and knowledge dynamics applies to any kind of discourse. Fourth, *thought patterns to a considerable extent consist of so-called conceptual metaphors*. They should be understood as a cognitive deep structure aggregated from converging linguistic metaphors – to be understood as surface structures (Lakoff 1993: 244) – which can be analytically summarised in so-called idealised cognitive models (Lakoff 1987: 68), or models of thought (Weinrich 1976: 294). Finally, *metaphors are based on a cognitive mapping process*: “A metaphor [...] is a process by which we understand and structure one domain of experience in terms of another domain of a different kind” (Johnson 1987: 15). Hence, more concrete meanings (source domain) are projected via a mapping upon an abstract domain (target domain) to make it semantically, cognitively and practically accessible. This mapping (Weinrich 1976: 283) creates the possibility to analytically access and assess possible implications carried over from the source to the target domain. This can uncover “[…] implication complexes […]” (Black 1993: 28) enabling a wide range of possible associations which the focussing aspect of metaphor semantically compresses. Although compressed, implication complexes are important because the metaphorical mapping transfers meaning and connected moral values which require linguistic attention (Johnson 1993) and ethical inspection (Kaebnick and Murray 2013).

All these aspects of metaphors are interesting in a context where a certain stagnation in metaphors used in the discourses about genetics, genomics and post-genomic research has been found (McLeod and Nerlich 2017: 6). So-called canonised metaphors such as “books”, “maps”, “blueprints”, “programmes” or a mechanistic imagery (Boudry and Pigliucci 2013) are still used to scientifically conceptualise and publicly convey different sorts of meanings – and moral implications – about scientific issues, progress in research and their ethical, legal and social dimensions (Hellsten and Nerlich 2008; Nerlich and Hellsten 2009; Nerlich et al. 2009). This development also received criticism by synthetic biologists such as Porcar and Peretó (2016) or de Lorenzo (2011) (see also de Lorenzo (in press), this thematic series). They acknowledge the heuristic potentials and the semantic traps inherent in different kinds of metaphors. In their view, “metaphorical biology” (Paton 1992) – that is a biological research conscious about its metaphorical status – requires a critical inspection of the implications nestling in metaphors and conceptual images, to enable reflexive and responsible use of images in scientific thinking, practice and public communication.

The metaphors of “writing”, “books”, “reading” or “blueprints” became conventionalised metaphorical reservoirs (Kueffer and Larson 2014: 720) through which they convey the image that the chemical structure of the DNA is information that can be read, understood and – to use another metaphor – be re-written, indeed edited.

Going beyond such prior research, this paper develops a methodological approach that helps to partly reveal the metaphorical production and transmission of morality in the German news coverage. Studying the moral implications of metaphorical mappings in this way is important because “[...] the way we frame a given situation [or new technology] will determine what we ought to do about it, and our semantic frames [...] are – at least to some extent – based on metaphor” (Johnson 1993: 52).

## Metaphors in synthetic biology: a method for analysing moral implications

**Fig. 1:**
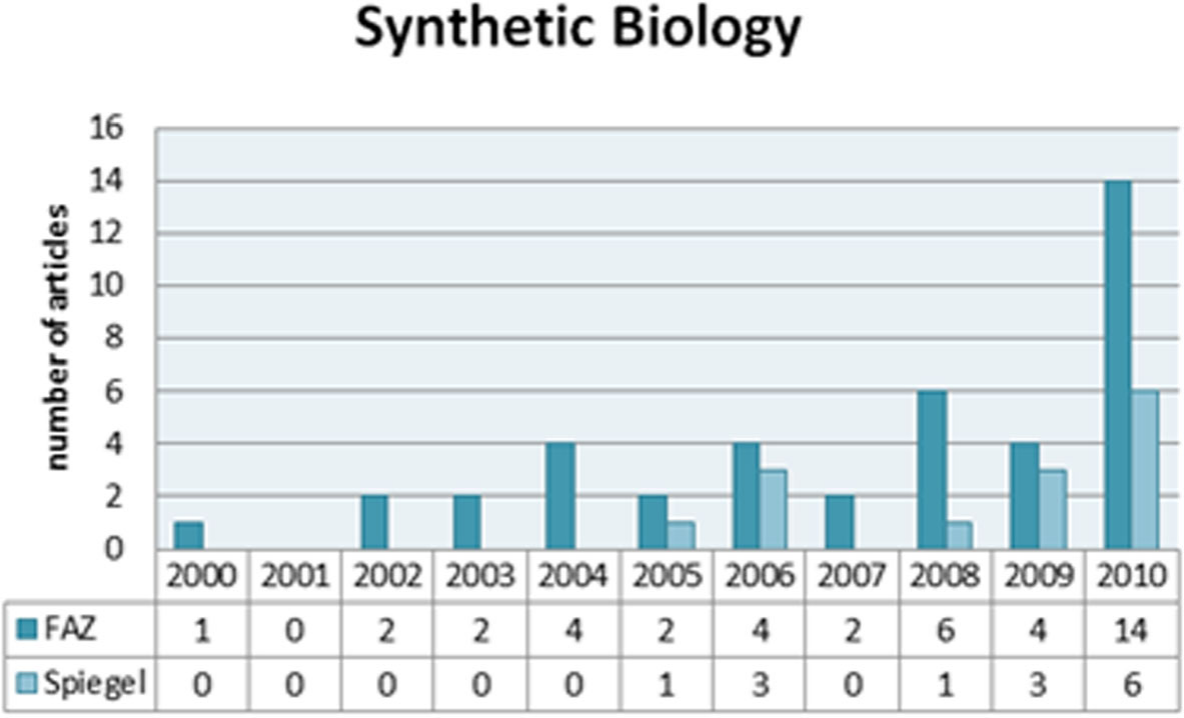
Number of articles appeared per year (kindly provided by Anne Brüninghaus)

A first step for analysing the news coverage consisted in establishing a corpus of newspaper articles about SynBio published in *Die Frankfurter Allgemeine Zeitung* and *Der Spiegel*. This was done using the LexisNexis news database for the period between 2000 and 2010 (Fig. 1).

This period was chosen as it represents the emerging and consolidation phase of SynBio and ends with the press conference given by Craig Venter on his newly designed artificial organism Mycoplasma Mycoides JCVI-syn1.0. The search term ‘Synthetische Biologie’ revealed an overall number of 41 articles for *Die Frankfurter Allgemeine* and 14 for *Der Spiegel*. All articles vary in length due to the category of print medium they appeared in and were mostly found in the news and the science section.

After having established the data base, the next methodological step consisted in developing a combined approach that merged linguistic (Jäkel 1997: 141–146) and science and technology studies’ (Maasen and Weingart 2000) perspectives of analysing metaphor. The approach consisted in a first reading of all newspaper articles which helped to get a contextual overview over the structure of the media corpus. First impressions, authors, institutions mentioned, science-policy aspects, visual images, different text types and themes covered were noted in a ‘minute book’ and later on systematised in form of a thematic map. Against this structural and contextualising background, a close line-by-line reading revealed all linguistic metaphors occurring in the two newspapers. This procedure involved a first analysis of the mapping processes to secure the metaphorical content of each metaphorical instantiation. Two hundred ninety-eight metaphorical expressions were found in the newspaper coverage and transferred into a table where creative or idiosyncratic metaphors were deleted and the 265 metaphorical expressions (he constructs protein molecules for genes, for example) and their mappings were re-analysed. This procedure enabled the exploration of the degree of metaphoricity of each metaphorical expression found and at the same helped to assess the source domains involved in the metaphorical mapping (building plans for proteins – for the example provided). Hence, the mappings of the whole corpus underwent an analysis that considered source and target domain as well as a reflection of the mapping processes involved. This procedure was followed by grouping the metaphorical expressions according to the mapping processes into higher-order conceptual metaphors. These conceptual metaphors represent generic structures that semantically permeate the news coverage and their recurrence bears a considerable impact on the overall meaning of the news coverage. Finally, the mappings in the linguistic metaphors underlying the different conceptual metaphors were analysed.

The novel aspect of the present approach resides in (a) examining the words surrounding the words used in the source domain using *Der Duden* (the German equivalent of the *Oxford English Dictionary*) and (b) consulting the co-occurrence database Cyril-Belica (corpora.ids-mannheim.de) where each word in a large German language corpus is listed with its ‘profile’ of words with which it regularly co-occurs, that is, with which it is frequently used together. Using this database revealed a host of connotations defining the intangible semantic field and implication complexes of each metaphor. This enabled me not only to define the exact meaning of each word in the source domain but also provided the possibility to reveal some normally elusive implications. These were systematised and transferred into a list for each conceptual metaphor, with a particular focus on moral implications. As a final step, the implications of each conceptual metaphor were discussed in view of responsible language use in terms of whether they provide problematic or fruitful ways of engaging with SynBio (Kueffer and Larson 2014: 722). This involves making some subjective judgements. However, these are based on long-standing discussions with colleagues about responsible language use in genomics and post-genomics (see Döring 2014). They are also grounded in what Nordgren (1998) calls imaginative ethics and where the key “that morality has to do with metaphors and imagination rather than with well-defined concepts and deduction” (p. 117).

In sum, this linguistically informed qualitative approach tries to pave the way for a more systematic and empirical way to reveal moral implications inherent in metaphors. In doing so, it aims at providing a small step towards a more grounded analysis of moral implications and their ethical problematisation. I will now turn to four prevailing conceptual metaphors derived from the 298 metaphorical expressions found in the media coverage, in which “metaphor works by applying to the principal subject a system of associated implications characteristic of the subsidiary subject [...]” (Black 1993: 28).

## Metaphors and morality in the German coverage of SynBio

The media discourse in *Frankfurter Allgemeine Zeitung* and *Der Spiegel* is mainly characterised by four overlapping conceptual metaphors: doing synbio is constructing, doing synbio is playing a game, doing synbio is programming and doing synbio is writing and editing a text. In the following, each conceptual metaphor will be illustrated by paradigmatic linguistic metaphors taken from the corpus.

To start with, the most prominent conceptual metaphor found in the media corpus is doing synbio is constructing. Here, the linguistic metaphors found are mainly informed by the verbs “bauen” (to build), as in the following example:

1. Die Industrie brauche aauch künftig hervorragend ausgebildete Chemiker, um die für die Produktion von Substanzen notwendigen **Moleküle “bauen” zu können**. (FAZ 29.10.2002: 49) (Even in the future, industry needs excellently trained chemists who can “build” the necessary molecules for the production of substances)

The verb “to build” appears here in the context of a newspaper article providing information about an early workshop for students studying SynBio. The metaphorical mapping inherent in the phrase “building molecules” (Moleküle bauen) is taken from the more general meaning of “bauen” and clearly applies the semantic field of building construction. The mapping applies what people know about building sites, bricklayers, bricks and all processes involved in house construction to the scientific fields of SynBio. In the quote, the chemists mentioned are – metaphorically speaking – bricklayers on molecular construction sites, and the analysis of “bauen” in the dictionary *Der Duden* reveals implications of a deliberate and systematically plan carried out. The co-occurrence database reveals that the verb “bauen” is strongly correlated with all sorts of buildings and vehicles, which corroborates aspects of a structured and systematic approach which results in producing something.

As building involves the use of vehicles, in the following quote an allusion to our everyday knowledge of vehicles is made through the use of the slang adjective “aufgemotzt” (pimping a car):

2. Wenn sie das Klassenziel erreichen, haben die Studenten **eine Lebensform konstruiert**, die es so nicht gab: einen **synthetisch aufgemotzten Organismus** […]. (FAZ 07.03.2004: 65) (If they attain their group’s aim, the students have constructed a life form that didn’t exist before: a synthetically pimped organism)

Here, the metaphorical mapping transfers semantic aspects of tuning cars to genetically modified organisms and helps to convey a meaning of technical improvement as well as an aesthetical change regarding the appearance of the object. The textual context, however, restricts the semantic scope of the source domain to cars and car production – which could also be thought of as the metaphor of the chassis in SynBio – and communicates rather technical implications of production and improvement. This aspect is also corroborated by the findings in the co-occurrence database, in which the semantic field of “konstruieren” (to construct) mainly revolves around vehicles, clocks and –astonishingly – genetic engineering. This result indicates that the meaning of “konstruieren”, at least in the German language, has begun to connotate aspects of genetics and biotechnologies.

Comparable features also emerge in the following case, where the previously mentioned source domains of construction and vehicles are exploited in the nouns “Vehikel” (vehicle) and “genetische Konstrukte” (genetic constructs):

3. […] gibt es eine Alternative, bei der man gleich ganz auf natürliche **Vehikel für genetische Konstrukte** verzichtet. (FAZ 07.03.2004: 65) (there is an alternative where one can do completely without the natural vehicle in order to make genetic constructs)

The noun “Vehikel” (vehicle) metaphorically maps the technical aspects of cars or other means of transport onto the domain of biological carriers, while the notion of genetic constructs refers to genetically engineered entities. Following Cyril-Belica, the metaphorical use of vehicle clearly entails a semantic dimension of car driving, motorisation and traffic, while the notion of construct – as in the case of the verb “to construct” – inherits meaning dimensions revolving around biology and genetics.

Meanings of building and constructing also have an impact on the use of other nouns. The metaphorical use of modules and bricks is quite frequent in the corpus, either in compounds such as “Genbausteine” (genetic building blocks) or “Bausteine” (building blocks).

4. Sie wollen nicht nur hier und dort ein Gen von Tier zu Tier transferieren oder auf gut Glück den einen oder anderen **Genbaustein austauschen**. (Spiegel 01/2010: 113) (They don’t just want to transfer a gene here and there from animal to animal or randomly exchange this or that genetic building block)

“Genbaustein” appears noticeably often and the notion of building block is clearly defined in *Der Duden* as stones for building. The entries found in *Cyril-Belica*, however, indicate that the meaning of building block has been affected by its metaphorical use in biology as frequent co-occurrences such as cell membrane, decoding or amino acid indicate. Hence, the language is beginning to change through what one might call ‘genetically modified metaphors’.

While meanings of construction and vehicles are changing in German under the influence of biology, SynBio is still dominated by meanings derived from ordinary uses of construction and engineering. But is the building or constructing of new forms of life in terms of cells or metabolic pathways really ‘engineering’? Proponents of responsible language use would question this easy mapping of construction and engineering onto SynBio and ask about the release of previously non-existent forms of life and its ecological consequences for bio security and bio safety, for example, as well as the deterministic connotations of engineering.

The conceptual metaphor of doing synbio is playing a game is closely related to its partner doing synbio is constructing. Both converge in the fact that the words of building bricks and toy blocks are semantically related. The way towards this relationship in the context of SynBio has been paved by the use of metaphors by Drew Endy for example:

5. Forscher haben begonnen, **Mikroben nach dem Baukastenprinzip neu zusammenzusetzen**: Die Kunst-Organismen sollen Rohstoffe produzieren… […]. (Spiegel 33/2006: 126) (Researchers have begun to put together microbes according to the construction kit principle: these artificial organisms are supposed to produce raw materials)

Here, the metaphorical transfer of constructing has been mapped onto a specific children’s toy, the “Baukasten” (construction kit – in Germany normally consisting of coloured wooden blocks in a box). *Der Duden* explicitly refers in its entry to model kits to toy blocks, while the database *Cyil-Belica* strongly points towards the notion of Lego bricks as a toy. Hence, a new dimension of meaning enters the scene: playfulness which is conceptually linked with scientific work. Such aspects are clearly mirrored in the use of diminutives such as “Bauklötzchen” (toy bricklets) that highlight converging aspects of small size (genetic entities) and children’s games:

6. Mit **genetischen Bauklötzchen** und synthetischen Molekülen wollen Forscher künstliche Bakterien und Viren konstruieren. (FAZ 07.03.2004: 65) (Using genetic toy bricks and synthetic molecules, researchers want to construct artificial bacteria and viruses)

The metaphorical construct “genetic toy bricks” in the previous quote includes these aspects and implicitly links the childlike joy of playing with scientific creativity and tinkering. These aspects converge with the Lego-metaphor, which conceptualises scientists working in SynBio as “Lego-Bauer” (Lego-builders) (Spiegel 01/2010: 113), while their targeted genetic or modified entities become Lego bricks. This metaphor goes back to Drew Endy, a well-known proponent of SynBio, and has been described by *Der Spiegel* as follows:

7. Als Kind begeisterte sich Endy für die unerschöpflichen Möglichkeiten der **Lego- Steine**, und **Lego-Bauer** ist er im Grunde geblieben. Nur dass er seine Werke nun aus anderen Bausteinen errichtet: Er bedient sich des **Baukastens der Natur**. (Spiegel 01/2010: 113) (As a child Endy was excited by the inexhaustible possibilities of Lego bricks; and he still remains a Lego builder. Of course, he now constructs his works of art out of different bricks: He uses the construction kit of nature)

The quote tells the story of Endy’s fascination with the unlimited potentials of Lego bricks and elaborates on this analogy by metaphorically depicting him and fellow scientists as Lego-Builders (Lego-Bauer) who use nature as their construction kit (“Baukasten der Natur”), and ever since, this metaphor has been used to conceptualise newly developed genetic entities. These entities metaphorically became – in allusion to Watson and Cricks model of the double helix – coloured Lego bricks:

8. **Bunten Legosteinen** gleich werden dabei genetische Bauteile eingesetzt, um Bakterien wunschgemäß besondere Eigenschaften zu verleihen […]. (FAZ 27.01.2008: 61) (Similar to coloured Lego bricks, genetic construction pieces are used to endow bacteria with specific desired attributes)

The metaphorical use of Lego led to the idea of a “Lego Biologie” (FAZ 12.11.2008: N1) which represents “synthetisches Bastelmaterial” (synthetic craft materials) (FAZ 26.05.2010: 29). Hence, the source domain of Lego and the metaphorical mapping functioned as an all-encompassing semantic reservoir to conceptualise genetic entities, the process of construction, the scientific discipline that focuses on this construction and the actors doing the construction work.

A look in *Der Duden* shows that there is no entry for Lego while the database Cyril Belica exhibits co-occurrences such as “Spielzeughersteller” (toy manufacturer), “Spielsachen” (toys; literally: ‘play things’) or “Baukasten” (toy kit). The semantic focus here is on playfulness, childhood play and creativity. The question, however, remains as to whether the development of artificial forms of life should be framed in this playful and euphemistic way. The connotations and implications of Endy’s metaphor brush over problematic aspects of SynBio and convey ideas of innocent playing and tinkering with toys which, in the context of SynBio, should however be based on stringent bio security measures. Hence, the metaphor opens up a network of implications that responsible language use should unmask and critically discuss.

The third metaphorical concept used is the conceptual metaphor doing synbio is programming. Here, images revolving around computers and software are mainly communicated through verbs such as “programmieren” (programming) and “umprogrammieren” (reprogramming). Biologists, geneticists or chemists become computer programmers, while cells and other biological entities such as metabolic processes are conceived as computers or software. Hence, interventions in cells and other biological entities are framed by mappings derived from the source domain of information technology:

9. Von ihr erwartet man eine Vielzahl von biotechnischen Umbrüchen - beispielsweise **Bakterien, die so programmiert werden**, dass sie Kohle in Biogas umwandeln, oder Mikroben, die Kerosin produzieren. (FAZ 15.08.2009: 31) (We expect from it a multitude of biotechnical breakthroughs – for example bacteria which are programmed in such a way as to transform coal into biogas, or microbes that produce kerosene)

*Der Duden* clearly lists an information technology focus on the verb “programmieren” (programming), and this is corroborated by the search on the co-occurrence database: the metaphorical mapping transmits an information technology focus. The same also holds true for the verb “umprogrammieren” (reprogramming), which refers to a change in the functioning of a biological entity:

10. Wenn dem so ist, wäre das eine gute Nachricht, denn Venter und sein Team sind gegenwärtig dabei, mit Forschungsgeldern von Exxon-Mobil eine fünf bis sieben Quadratkilometer große Algenfarm einzurichten, in der **umprogrammierte Algen Biokraftstoff produzieren werden**. (FAZ 15.08.2009: 31) (If this were true, it would be good news, as Venter and his team are currently engaged in creating an algae farm of seven square kilometres with money from Exxon-Mobil, in which reprogrammed algae will produce biofuel)

The semantic focus on information technology also affects the images used to conceptualise different levels of biological entities and their prescribed functions. In the following quote, for example, journalists go as far as to compare bacteria to universal Turing machines:

11. Es ist durchaus üblich, Bakterien mit universellen Turing-Maschinen zu vergleichen, **Zellen sind dementsprechend Computer und DNA eine Software**. (FAZ 21.05.2010: 33) (It’s absolutely common to compare bacteria with universal Turing machines; accordingly, cells are computers and DNA is software)

Hence, the metaphorical mapping envisages cells as computers and DNA as software. It cognitively provides the opportunity to act upon biological processes from a different conceptual angle than biologists have been accustomed to do. This interpretation is also mirrored in the patterns of IT-related co-occurrences in the *Cyril-Belica* database: Prevalent nouns such as “hardware”, “software”, “engineering” etc., tighten the semantic field and set a precise focus reconfiguring the semantics of genetics and biology.

Computer programs do not only require reprogramming in general, they also need a clear description of how to execute the instructions of a computer programme. Here, the old metaphor of “schreiben” (writing) comes into play, but in the sense of writing programmes rather than letters on a piece of paper. From there, extrapolations are made to ‘guiding’ not only nature but also evolution:

12. Irgendwann, so verkünden die kühnsten der Visionäre, würden sie auch **genetische Programme schreiben**, mit denen sich nicht nur die Natur, sondern sogar **die Evolution des Menschen selbst steuern ließe**. (Spiegel 01/2010: 113) (Sometime in the future, announce the most daring visionaries, they’d also write genetic programmes with which one could guide not only nature but the evolution of humankind itself)

The metaphor strongly resonates with the images used in the news coverage about the decoding of the human genome. Though not explicitly mentioned, DNA is here envisioned as the basic program and the strong deterministic thinking implicated in it leads to the conclusion that even the evolution of humankind could be controlled one day. The network of implications associated with the writing metaphor found in *Der Duden* and in *Cyril-Belica* strongly connotes traditional meanings as well as elements nowadays typical of IT-technologies. But a future step is at hand for so-called “gene engineers”, namely “blind writing”, which does not require programmers but works on the basis of automated and autonomous algorithms:

13. Die Geningenieure von morgen sollen daher den Computer Zufallsauswahlen kombinieren lassen, **schreiben quasi blind die Software synthetischer Organismen**. (Spiegel-Online 27.12.2008) (The gene engineers of tomorrow are therefore supposed to let the computer combine random selections, to write quasi blindly the software of synthetic organisms)

All in all, the metaphorical reservoir of IT-technologies frames biological processes, entities and their modification as a computational/technological endeavour. This conceptualisation is problematic as it focuses on the linear manipulation of life processes and, furthermore, transmits ideas of control of isolated products or processes which hide biological complexity. The narrowness of this conceptual metaphor therefore not only necessitates reflection when used, but also an explicit negotiation and assessment of its underlying assumption: Is doing biology really engineering, programming and reprogramming?

The aforementioned aspects and implications partly converge with the conceptual metaphor of doing synbio is reading and writing which is the last metaphorical concept examined here. This well-known metaphor was used in the context of SynBio to introduce a shift from reading to writing and indicates a conceptual and epistemic turning point from understanding to creation. Consequently, the metaphorical use of “entschlüsseln” (decoding), “lesen” (reading) and “Entschlüsselung” (deciphering) prevail in the first segment of the corpus because the development of SynBio was still ongoing and the decoding of the human genome only happened a few years before. The journalist actually refers to the “success metaphor of ‘deciphering”:

14. Bisher habe man den **genetischen Code gelesen**. Nun gelte es, **ihn zu schreiben**. So greift Venter die Erfolgsmetapher von der “**Entschlüsselung**” des Genoms und die damit **verbundene Erwartung einer Lesbarmachung des Erbgutes auf**. (FAZ 25.05.2010: 29) (Up to now we have been able to read the genetic code. Now we are supposed to write it. This is how Venter reconfigures the success metaphor of the decipherment of the genome and the expectation that’s associated with it of making hereditary genetic material readable)

This quote brings together all these prevailing images and their well-known metaphorical mappings. A close inspection of words like “decipherment” in Cyril-Belica proves that all these metaphors have nowadays been taken over by genetics as many co-occurrences with “Erbgut” (genotype), “Genom” (genome) or “Gen” (gene) indicate. Furthermore, these metaphors with their “genetische Buchstaben” (genetic characters) (FAZ 2701.2008), their “Textsalat[e]” (text salads) and their “Textfetzen” (scraps of text) (both Spiegel 01/2010: 110–111) paved the way for the shift from understanding to writing:

15. “Mit der **DNA schreiben wir die Anleitung**”, sagt George Church in seinem Eckbüro in der Abteilung für Genetik, “und **programmieren die Zelle wie einen Computer**. “(Spiegel 33/2006: 127) (“Using DNA we write the instruction”, says George Church in his corner office in the department of genetics,” and we programme the cell like a computer”)

The source domain of writing enabled a conceptual shift from understanding to creating. It was also flexible enough to be reused and re-metaphorised in metaphors of computational and engineered writing, re-writing and – metaphorically speaking – creative writing. The last aspect in particular is mirrored in the final quote of this section, in which the aim of SynBio is expressed in “deren Genom neu geschrieben werden soll” (whose genome will have to be newly written):

16. Im Gegensatz zu der “Bottom-up”- Konstruktion geht dieser “Top-down”-Ansatz von einfachen Bakterien aus, **deren Genom aber neu geschrieben werden soll**. (Spiegel-Online 09.07. 2006) (In contrast with the “bottom-up” construction, this “top-down” approach starts with simple bacteria, whose genome is however supposed to be re-written)

A look at both *Der Duden* and *Cyril Belica* shows that words like “reading” and “writing” are still firmly rooted in the semantic field of books and texts. Their meanings have not yet extended to uses in genomics or post-genomics. The verb “entschlüsseln” (deciphering), by contrast, has now acquired a strong semantic focus on genetics, genotype and genes. This has implications for responsible language use, as it is almost impossible to avoid these ‘metaphors’. They have become basic and almost unavoidable lexical and conceptual tools for talking about genomic and post-genomic innovation and technologies.

**Fig. 2:**
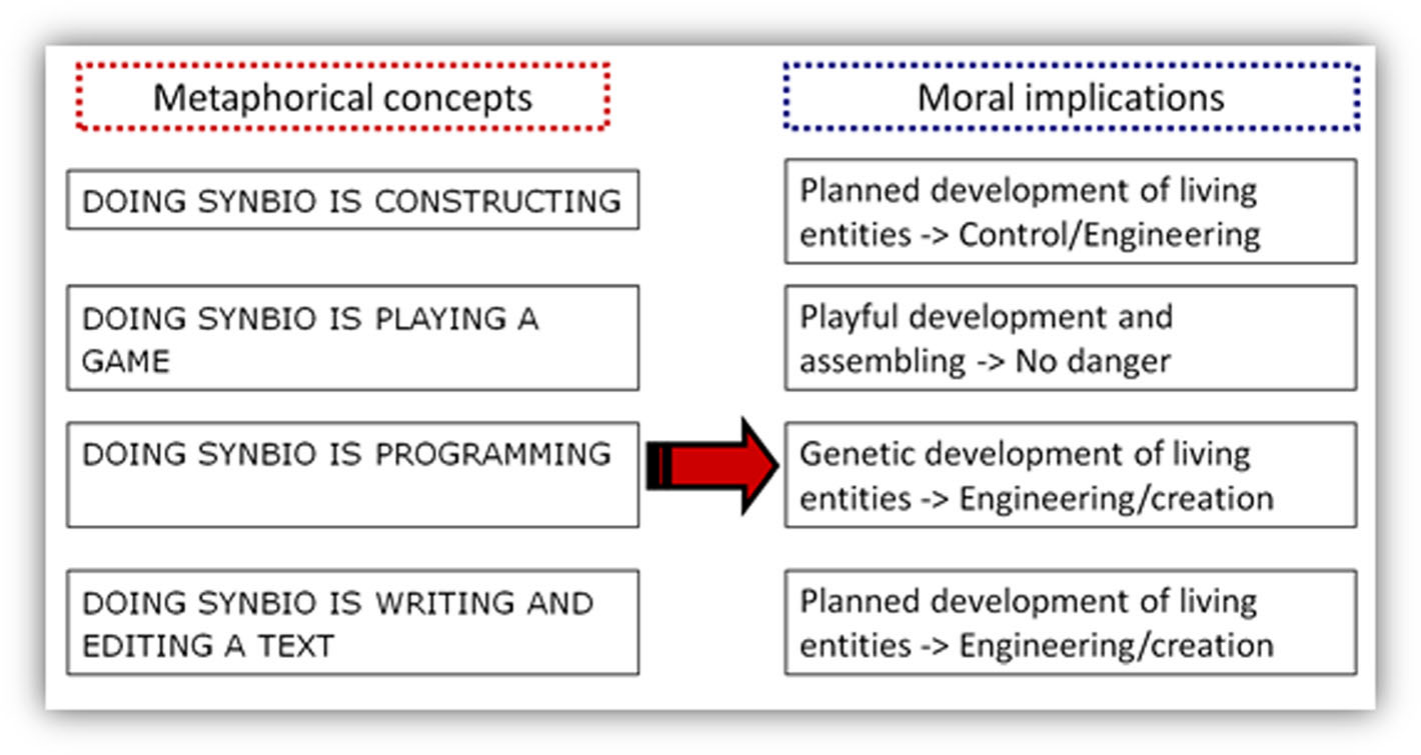
Moral implications of media-metaphors

To conclude, the analysis revealed four prevailing conceptual metaphors and the study of the implications nestling in the media-metaphorical mappings uncovered the different and sometimes converging arrays of moral implications inherent in these metaphors. These are summarised in the following table (Fig. 2).

Overall, the conceptual metaphors used by the German media highlight aspects of controlled engineering (Cserer and Seiringer 2009) on the one hand and playful, even innocuous, scientific work on the other. Although critical comments with regard to problems revolving around biosafety and biosecurity are occasionally mentioned in the news coverage, journalists (and the scientists they quote) only rarely address the possible dangers, impacts and implications of this technology for biology and society. This can be seen as a lack of responsible thinking induced by irresponsible language use.

The newsworthiness of a revolutionary technology and scientific discipline appears to determine the semantics of the news coverage between 2000 and 2010, while a responsible use of metaphors and their possible implications is simply lacking and this constrains responsible thinking as well. Consequently, ethical, legal and social implications of SynBio stay in the background while positive visions of scientific progress abound, creating great expectations of biomedical remedies for modern diseases, combating hunger, making new types of fuel or simply promising genetically modified bacteria that can ‘eat’ ocean oil spills.

## The language of SynBio: between hidden moralities and responsibility

Between 2000 and 2010, two seminal news outlets in Germany depicted Synbio mainly through the lens of four conceptual metaphors that create an unproblematic and technocratic image of SynBio. The semantic associations analysed with the help of *Cyril-Belica* revealed hidden moral implications inherent in the metaphorical mapping processes. The semantic analysis of the words used in the source domains hence proved to be useful as the target domains are not only semantically structured by them, but also inherit the implications and value complexes connected with the metaphorical use of the lexical items used. This not only shows how abstract entities or aspects are framed by concrete domains, cultural knowledge or experiences, but how the source domain resonates with intangible semantic fields and values, and projects its implications onto the target domain.

This hidden moral aspect of metaphor use has to date not systematically been studied in this way, neither in research on metaphor (Johnson 1993; Lakoff and Johnson 1999), nor in its role in science technology studies, the social study of science or the sociology of scientific knowledge. The theoretical and empirical potentials outlined here require further conceptual and empirical elaboration. This also applies to the method used.

The findings of this paper support a call for more research into the responsible use of language in general and of metaphor in particular (Kueffer and Larson 2014).

A technology assessment as metaphor assessment (Mambrey and Tepper 2000), as it has been carried out in this article with respect to media coverage, creates awareness of the moral implications of unconscious conceptual metaphors and can increase understanding of how public opinions may be shaped by them. It can, of course, also facilitate ethical reflection and negotiation in science and society. This would, however, require further innovation in terms of enabling participative processes, which avoid the deficit model of public understanding of science and in which scientists, journalists and audiences collaboratively assess the emerging implications of metaphors (Larson 2011). Indeed, this applies to scholars, policy makers, industry and the public who all are – willingly or unwillingly – involved in the project of SynBio. Whatever linguistic structures or images are used, the language of SynBio and the social, ethical, legal and political associations it carries requires constant scrutiny because – to paraphrase Abraham Lincoln – you cannot escape ethical responsibility for technologies by evading their moral implications.
